# An evidence-informed approach to address food and nutrition security: the ecology of infant feeding practices

**DOI:** 10.3389/fped.2023.1212607

**Published:** 2023-09-12

**Authors:** Daniel J. Raiten, Andrew A. Bremer

**Affiliations:** Pediatric Growth and Nutrition Branch, Eunice Kennedy Shriver National Institute of Child Health and Human Development, National Institutes of Health, US Department of Health and Human Services, Bethesda, MD, United States

**Keywords:** human milk, infant feeding, dietary guidelines, national strategy, nutrition ecology

## Introduction

There has been increasing attention paid to the need for a deeper understanding of those factors that contribute to the daunting level of food and nutrition insecurity in the US and globally. The ability to achieve the aspirations of these efforts requires a life course approach that begins with the critical developmental period referred to as the “First 1,000 Days” encompassing pregnancy through the first 2 years of life. Multiple global health targets have been disseminated to address this critical period and most directly the efforts to develop and sustain resilient approaches to infant feeding (1).

In the US, the historical interest in the promulgation of evidence-informed guidance was reinforced most recently by a series of activities aimed at integrating the 1,000 days period in the US Dietary Guidelines for Americans (DGA). These efforts were stimulated by a project that ultimately became known as the “B-24 Project” aimed at evaluating the evidence to support the integration of infants and children from birth to 24 months into the DGA (2). Due to the tremendous advocacy of the US government (USG) public health community, dietary guidance during pregnancy and the first two years of life was included in the 2020–2025 DGA and will be fully integrated into all future DGA iterations. In addition, since the finding that a critical gap exists in our knowledge about the factors affecting human milk composition (3), the USG community has initiated a concerted effort to compile existing and generate new data to address this need (4).

### The *Eunice Kennedy Shriver* National Institute of Child Health and Human Development (NICHD)

Since its inception 60 years ago, NICHD has supported research in the nutrition and infant feeding spaces. More recently, NICHD has further codified its mission with the publication of its Strategic Plan (5), which highlights the critical and cross-cutting topic of nutrition and, in particular, the importance of an expanded understanding of the complexities of the inherent biology and implications of factors affecting decisions and implementation of best practices to support nutrition, health, and development via context-specific equitable, safe, and efficacious infant feeding practices.

### The 2022 White House Conference on Hunger, Nutrition, and Health

In September 2022, the Biden-Harris Administration released its National Strategy on Hunger, Nutrition, and Health (referred to as the “National Strategy”) and the Administration hosted the White House Conference on Hunger, Nutrition, and Health, both organized around five pillars that highlight the continuum of activity across all agencies that directly or indirectly impact food and nutrition insecurity and diet-related diseases and the need for an evidence base to support these efforts (6) (Box 1). In addition, these pillars are illustrative of a recognition that research and translational efforts demand a deeper understanding of the full spectrum of issues impacting on the biology, choice, and implementation of context-specific, equitable, safe, and efficacious programs, policies, and standards of care.

**BOX 1:** The National Strategy: organizing pillars

• Pillar 1 – Improve food access and affordability: end hunger by making it easier for everyone – including individuals in urban, suburban, rural, and Tribal communities and territories – to access and afford food. • Pillar 2 – Integrate nutrition and health: prioritize the role of nutrition and food security in overall health – including disease prevention and management – and ensure that our health care system addresses the nutrition needs of all people. • Pillar 3 – Empower all consumers to make and have access to healthy choices: foster environments that enable all people to easily make informed, healthy choices, increase access to healthy food, encourage healthy workplace and school policies, and invest in public education campaigns that are culturally appropriate and resonate with specific communities. • Pillar 4 – Support physical activity for all: make it easier for people to be more physically active – in part by ensuring that everyone has access to safe places to be active, increase awareness of the benefits of physical activity, and conduct research on and measure physical activity. • Pillar 5 – Enhance nutrition and food security research: improve nutrition metrics, data collection, and research to inform nutrition and food security policy, particularly on issues of equity, access, and disparities.

### The “Breastmilk Ecology: Genesis of Infant Nutrition (BEGIN)” Project

The complexity of this new understanding is exemplified by the myriad factors to consider in addressing infant feeding practices, including human milk feeding. Because of the inherent complexity of that new understanding, a new approach is required that recognizes that these issues represent an “ecology” in which human milk is a complex biological system affected by both internal (biology, genetics, nutrition, health) and external (social determinants, food system, physical) environments. The BEGIN Project – initiated by NICHD in 2020 – was incepted to apply this ecological framework to our efforts to support and expand the infant feeding agenda in the US and globally.

## Discussion

Human milk is the gold standard source of nutrition and other critical bioactive components to support the growth and development of infants. It is, in most circumstances, the best source of nutrition and serves as nature's barrier against food insecurity for infants. Breastfeeding remains the primary mode of delivery of human milk. However, our ability to achieve a greater prevalence of exclusive and extended breastfeeding based on accepted guidance remains a challenge, particularly for underserved populations in the US and globally. Moreover, due to changing social and economic environments, new modes have emerged as options for parents wanting to provide human milk to their babies, including feeding of expressed milk and, in cases where the parent's own milk is not available, use of donor/banked human milk. In addition, in cases where neither parent's own milk nor donor milk is a viable option, a need exists for safe and efficacious human milk substitutes. Our ability to make evidence-informed recommendations/guidance about various aspects of infant feeding practices and related aspects of the determination of nutritional requirements for infants thus demands a deeper appreciation of the factors that affect the composition and delivery of human milk.

The BEGIN Project was designed to provide evidence with regard to a deeper understanding of human milk composition and its ecology (the interactions amongst the infant feeding triad, i.e., parental biology, the breastfeeding infant, and the unique nature of human milk as an interactive biological system affected by both an internal and external environment). The information derived from the BEGIN Project is intended to inform those agencies/organizations in the public and private sectors involved in the development and promulgation of evidence-informed guidance about safe and efficacious infant feeding practices and the development of a research agenda to support all aspects of those efforts (7). Hopefully through the efforts described above a stronger foundation can be built upon which to mount the programs and policies needed to fulfill the promise outlined by the White House Conference and those pillars to support the health and development of generations to come.
